# Contact-electro-catalysis at Dynamic Semiconductor–Water Junctions

**DOI:** 10.34133/research.0940

**Published:** 2025-10-07

**Authors:** Zhanqi Liu, Ziming Wang, Kaiyang Shi, Huifan Li, Yusen Su, Weihua Han, Zhong Lin Wang, Wei Tang

**Affiliations:** ^1^ School of Physical Science and Technology, Lanzhou University, Lanzhou 730000, China.; ^2^ Center for High-Entropy Energy and Systems, Beijing Institute of Nanoenergy and Nanosystems, Chinese Academy of Sciences, Beijing 101400, China.; ^3^ School of Nanoscience and Engineering, University of Chinese Academy of Sciences, Beijing 100049, China.

## Abstract

Junctions are the fundamental elements of semiconductor devices, which are crucial for advancements in modern technology. However, most junctions involve static solid interfaces. Here, we studied a dynamic Schottky-like junction formed at a semiconductor–water interface, referred to as a semiconductor–water junction (SWJ), under mechanical stimulation. We found that owing to the band bending at the junction, electrons can be transferred from water molecules to p-type silicon and captured by the dissolved oxygen molecules in water periodically, resulting in abundant free radicals and catalytic capacity for degrading pollution such as methyl orange. We demonstrated that contact-electro-catalysis is the underlying mechanism, and it can be applied to various SWJs. Furthermore, by replacing water with methanol aqueous solution, hydrogen can be catalytically produced with low energy consumption. We anticipate that SWJ-stimulated contact-electro-catalysis will provide a new space for innovative catalytic strategies in the future.

## Introduction

Semiconductors have been instrumental in advancing human civilization. They underpin transformative technologies, including integrated circuits, portable electronics, and the emerging artificial intelligence.

In addition, they enabled innovative strategies for lighting and display in a low-carbon manner via light-emitting diodes [1,2]. In recent decades, owing to the unique electronic structure and the coupling of crucial junctions with photons, the solar cell was developed, showing a shifting paradigm of new energy utilization and promoting green transformation of traditional fossil energy [3–5]. Meanwhile, a similar principle inspired researchers to propose photocatalysis, enabling emerging advancements in catalytic processes, such as water splitting [6–9], nitrogen fixation [10,11], and carbon dioxide reduction [12,13], contributing to sustainable development for the earth. To sum up, the electronic structure and diverse junctions of semiconductors markedly spawned the upgrading of various traditional fields.

Noteworthily, the interface between a semiconductor and a liquid also forms a junction, governed by the Mott–Schottky rule, proposed in the 1930s [14,15]. Water is the most commonly used solvent, and the charge transfer process across its interface has laid the foundation for various life and chemical processes. The static or quasi-static property of a semiconductor–water junction (SWJ) is critical for analyzing several vital parameters such as flat-band potential and doping density [16,17]. However, the characteristics under dynamic conditions have rarely been discussed. Here, we utilized mechanical stimulus to dynamically adjust the interface between a semiconductor and water, i.e., the SWJ, as well as its band bending status and depletion region. It was found that electrons and holes are periodically transferred across the interface, thereby catalyzing chemical reactions. Electron paramagnetic resonance (EPR) spectroscopy revealed that reactive oxygen species (ROS) were generated by the p-Si/water SWJ under vibrational conditions in the dark. Contact-electro-catalysis (CEC) has been proved as the dominant catalytic mechanism, and this phenomenon is feasible for a variety of semiconductors (such as GaN, Fe_2_O_3_, and InSb). The reactivity of the produced ROS enables it to degrade organic pollutants such as methyl orange (MO), and a low energy (0.21 W) consumption is required. Moreover, this SWJ-stimulated CEC can also be applied to the evolution of hydrogen from a methanol aqueous solution. The obtained evolution rate is maintained at 2.1 μmol/g/h/W for more than 10 h. These findings further exploit the catalytic effect of the semiconductor, especially on the surface, which gets rid of the recombination of electrons and holes inside. We expect that it will open new avenues for fundamental catalytic research, as well as innovative and sustainable development for green and low-carbon chemical industries.

## Results and Discussion

### Dynamic SWJs and ROS production

**Fig. 1.**
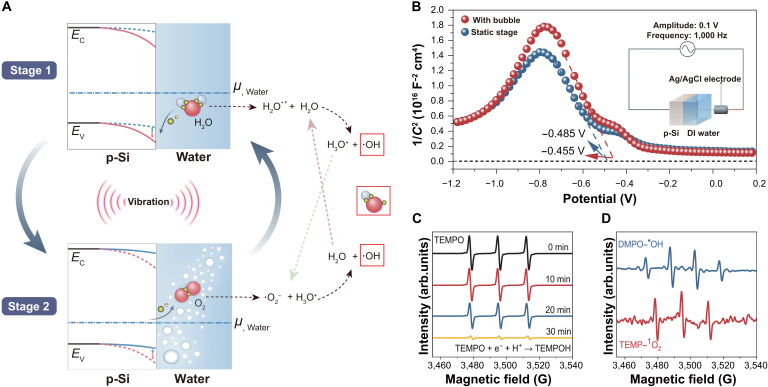
Investigation of the mechanism of a dynamic semiconductor–water junction (SWJ) and its catalysis effect. (A) Schematic illustration of energy band bending at a p-type silicon/water junction and the mechanism of radical generation. (B) Mott–Schottky plot of the junction between p-Si and deionized water. The inset shows the test circuit. (C) Detection of electron paramagnetic resonance (EPR) spectra for 2,2,6,6-tetramethylpiperidine-1-oxyl (TEMPO) at different time intervals. (D) Detection of the EPR spectra of 5,5-dimethyl-1-pyrroline *N*-oxide (DMPO) and 2,2,6,6-tetramethylpiperidine (TEMP) at 20 and 30 min. DI water, deionized water; TEMPOH, 2,2,6,6-tetramethylpiperidin-1-ol.

Figure 1A illustrates the energy band diagram between p-doped silicon (p-Si) and water. Due to the mismatch between the Fermi level of p-Si and the chemical potential of water oxidation, we hypothesize that electrons transfer from water molecules to p-Si upon their contact, forming an electron-rich region at the p-Si surface. Thus, as shown by the solid line in Fig. 1A stage 1, the energy band of p-Si will bend downward, forming an SWJ. A detailed diagram of the band bending from the initial contact is plotted in Fig. S1. We employed the Mott–Schottky test to measure this SWJ, and the results are shown in Fig. 1B (the inset presents the test circuit). The negative slope in the blue curve in Fig. 1B reflects the downward band bending at the semiconductor–water interface (static stage), with the equilibrium reached at −0.485 V. As illustrated in Fig. 1A (stage 2), mechanical perturbation was applied to the semiconductor–water interface, leading to the injection of bubbles. This transforms the local environment from a uniform water phase to a mixed air–water interface, altering the interfacial chemical potential. Consequently, the local volumetric ratio of water to air bubbles at the SWJ interface would decrease due to bubble injection, leading to a shift in the effective chemical potential on the water side and a reduction in the downward bending of the p-Si energy band, as observed in the red curve in Fig. 1B. The flat-band potential shifts to −0.455 V under bubble contact, as indicated by the red curve. Complementary open-circuit potential measurements confirm the modulation of band bending under mechanical perturbation (see Note S1 and Hurtado et al. [18]). Subsequently, when the bubbles disappear, the water-to-bubble ratio increases, and the SWJ returns to its initial state (Fig. 1A, stage 1), thus completing a full dynamic SWJ cycle. As the mechanical perturbation continues, the states of SWJ will vary periodically. Notably, the transfer of charge carriers in such cycles is capable of catalyzing chemical reactions. For example, during the first stage, electrons will be transferred to p-Si from water molecules, giving rise to the production of hydronium cations and hydroxyl radicals through a rapid proton transfer process [19,20]. At the second stage, owing to the variation of the chemical potential caused by bubbles, electrons will transfer from p-Si to the water side, especially to the dissolved oxygen to form superoxide radicals (·O_2_^−^), and ·O_2_^−^ is protonated into hydroperoxyl (HO_2_·) [21], leading to the formation of hydroxyl radicals by a chain reaction (Fig. 1A). To verify this mechanism, we placed p-Si powders in an aqueous solution of 2,2,6,6-tetramethylpiperidine-1-oxyl (TEMPO) and performed the reaction under dark and vibrating conditions. The EPR spectrum in Fig. 1C indicates a gradual weakening of the characteristic triplet peaks of TEMPO with the increase in reaction time, attributed to the formation of nonparamagnetic 2,2,6,6-tetramethylpiperidin-1-ol through electron and proton reactions involving TEMPO [22,23], verifying the occurrence of electron transfer between p-Si powders and water under vibrational conditions. The produczmethylpiperidine (TEMP) [24] and 5,5-dimethyl-1-pyrroline *N*-oxide (DMPO) [25] at different time intervals are illustrated in Fig. S2. The formation of ·O_2_^−^ and ·OH was also confirmed by using terephthalic acid (THA) and nitrotetrazolium blue chloride as captured in Fig. S3. Ab initio molecular dynamics simulations were further performed to validate this mechanism, and the details are available in Note S2. They suggest that the hole-rich p-Si facilitates the electron transfer from water molecules to the semiconductor, resulting in the formation of hydroxyl radicals in the interface.

### Catalytic degradation and material verification

**Fig. 2.**
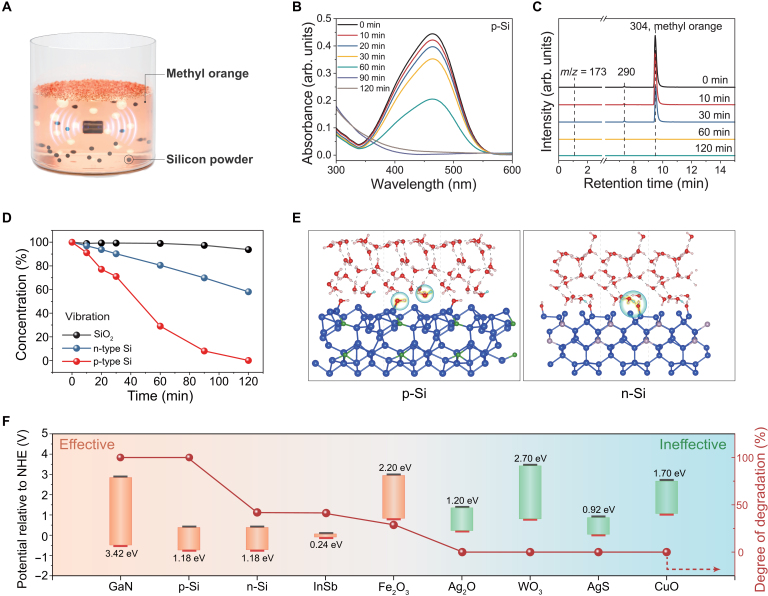
Degradation of methyl orange (MO) by dynamic SWJs under vibrational conditions. (A) Schematic illustration of silicon-powder-mediated MO degradation. (B) Ultraviolet–visible (UV–Vis) spectra of a 10-ml aqueous MO solution during vibrational conditions in the presence of 100 mg of p-Si powders for 120 min. (C) Mass spectral analysis of MO solution after separation by liquid chromatography at different reaction times. (D) Comparison of MO degradation by using SiO_2_, n-Si, and p-Si under mechanical vibrations. (E) The ab initio molecular dynamics (AIMD) simulation results of the p-Si/water and n-Si/water systems as they reach equilibrium. The blue, green, red, and pink spheres represent silicon, boron, oxygen, and hydrogen atoms, respectively. The hydrogen atom marked in sky blue represents the active protonic hydrogen. The circles highlight hydroxyl radicals and hydrated hydrogen ions. (F) Degradation of MO with various semiconductors; bandgaps are also marked in the figure (left: effective degradation semiconductors are marked in orange—GaN, p-Si, n-Si, InSb, and Fe_2_O_3_; right: ineffective degradation semiconductors are marked in blue—Ag_2_O, WO_3_, AgS, and CuO). The band positions of several semiconductors in contact with aqueous electrolyte at pH 0 [35–38]. The data represent the degradation results of MO after 2 h. NHE, normal hydrogen electrode.

The degradation of MO aqueous solution was employed as a model reaction to further verify the catalytic performance of the dynamic SWJ (Fig. 2A). The p-Si powder had a mesh size of 320, with a doping concentration of 1.1%. The mechanical stimuli were provided by a vibrator with a power of 0.21 W. Figure 2B indicates that the MO aqueous solution could be effectively degraded within 120 min under a dark condition. A control experiment was carried out in the same condition without vibration, showing almost no change (Fig. S4). A decrease in powder size (1,000 mesh) is associated with an improvement in degradation efficiency (Fig. S5), which could be ascribed to the enhancement of the contact surface area. We then examined the MO solution by liquid chromatography–mass spectrometry (LC–MS), as illustrated in Fig. 2C. The main peak of MO (retention time 8.61 min, *m*/*z* = 304) [26] gradually diminished, followed by new peaks forming around 290 and then decreasing with the reaction time, indicating that the discoloration of MO was due to the chemical reaction. Further analysis of the ultraviolet–visible (UV–Vis) and LC–MS spectra (Fig. S6) implies that the discoloration was due to oxidative degradation [27,28]. X-ray photoelectron spectroscopy (XPS) was carried out for p-Si powders’ examination before and after the reaction (Fig. S7). No new peak was observed. Additionally, we compared the morphology and elemental composition of p-Si powders before and after the reaction by scanning electron microscopy and energy-dispersive x-ray analysis (Fig. S8). All results indicate that the degradation of MO was mainly due to chemical degradation with p-Si serving as the catalyst. In addition, cyclic degradation experiments were performed to evaluate catalyst stability. The p-Si powder was reused for 3 consecutive cycles under vibrational conditions and consistently achieved complete degradation of MO (Fig. S9), confirming good reusability. Moreover, to demonstrate potential for practical wastewater treatment, we tested 3 additional common pollutants—rhodamine B, methylene blue, and malachite green—under the same conditions (5 ppm and 120 min). Complete degradation of all 3 dyes was achieved, as verified by UV–Vis spectra and visual inspection (Fig. S10).

As a comparison, we conducted the MO degradation experiment by using n-type silicon (n-Si) and SiO_2_ powders under identical conditions. The results in Fig. 2D show that the degradation of n-Si or SiO_2_ is much lower than that of p-Si. This implies that the native oxide layer on silicon will not be the dominant factor, and hole-rich p-Si will be beneficial for electrons transferring from water to semiconductor, thus resulting in free radical generation for MO catalytic degradation. The Mott–Schottky tests of n-Si with different doping concentrations (Fig. S11) indicate that the junction formed at the n-Si/water interface is opposite to that of p-Si. Furthermore, the corresponding depletion region is narrow, showing a small linear region. Ab initio molecular dynamics simulations confirm that, in contrast to the p-Si/water junction, the n-Si/water junction exhibits a much lower generation rate of hydrogen protons, which consequently leads to limited formation of hydroxyl radicals (Fig. 2E). Specifically, the hydrogen atom oscillates back and forth between the adsorbed water and other water molecules, as described in Note S2, without electron exchange. Therefore, the formation of hydroxyl radicals is suppressed (Fig. S12). Moreover, to demonstrate the universality of dynamic-SWJ-enabled catalysis, we selected various semiconductors for MO degradation, as shown in Fig. 2F. Among them, GaN, p-Si, n-Si, InSb, and Fe_2_O_3_ are effective for MO degradation (Fig. S13). Notably, the degradation rate does not show a clear correlation with the semiconductor’s bandgap. This suggests that the catalysis enabled by dynamic SWJ is distinct from photocatalysis, and the former is governed by the interfacial junction.

### Mechanical energy input investigations

**Fig. 3.**
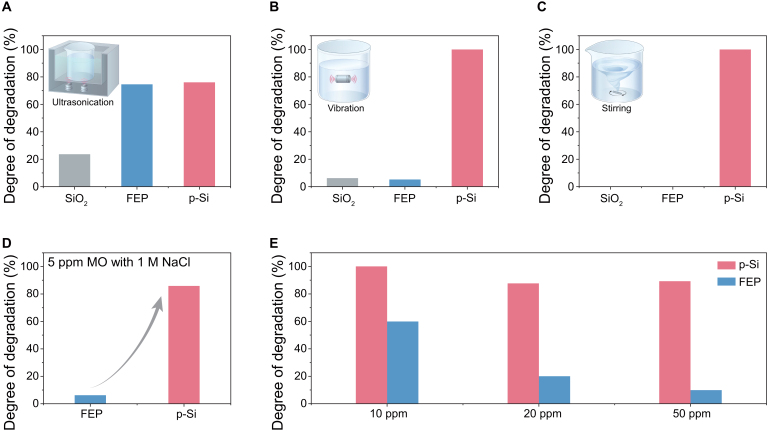
MO degradation results under different conditions. Comparison of MO degradation results for fluorinated ethylene propylene (FEP), p-Si, and SiO_2_ under conditions of (A) ultrasound (2 h), (B) vibration (2 h), and (C) stirring (6 h). (D) Comparison of MO degradation results for p-Si and FEP under ultrasound conditions in the presence of 1 M NaCl (4 h). (E) The influence of elevating MO concentrations on the degradation of MO by p-Si and FEP under ultrasound conditions (4 h).

We further investigated the degradation of MO under various mechanical stimuli, including ultrasonic waves, vibrations, and stirring. These experiments were designed to explore how different forms of mechanical energy influence the catalytic activity of semiconductor and dielectric materials. The detailed results are presented in Fig. S14. Under ultrasonic conditions, both fluorinated ethylene propylene (FEP) and p-Si exhibited a higher degradation rate compared to SiO_2_, as shown in Fig. 3A. SiO_2_ was included as a control because silicon surfaces may naturally oxidize, and previous studies have shown that silica can exhibit catalytic activity under ultrasonication [29]. More recently, SiO_2_ was also found to promote thiol oxidation via surface SiO· radicals at solid–water interfaces under ambient conditions [30], further highlighting its potential reactivity. Therefore, SiO_2_ helps rule out the influence of surface oxidation on the observed p-Si catalytic behavior. Additionally, under vibrational stimulus (Fig. 3B), p-Si displayed a significantly higher degradation rate for MO than FEP. In 2022, we introduced the concept of CEC, which is based on the electron transfer process during contact electrification [31]. These results indicate that SWJ-stimulated CEC is apparently different from dielectric-based CEC, which could be ascribed to the unique band structure of semiconductors. Furthermore, we employed magnetic stirring that delivers even lower mechanical energy for MO degradation. It is found that only p-Si demonstrated an obvious degradation as depicted in Fig. 3C, and a higher stirring speed results in an increased chemical degradation rate (Fig. S15). This result further indicates that even under mild mechanical input, the dynamic SWJ interface formed with p-Si can effectively catalyze interfacial redox reactions. Subsequently, MO degradation was conducted under 1,000 rpm stirring and air mass 1.5 Global (AM 1.5G) illumination (1 sun). Results show that degradation was enhanced in both the p-Si and n-Si groups compared to those without illumination (Fig. S16). Specifically, p-Si degraded 56.44% and 11.89% of MO within 120 min under illuminated and dark conditions, respectively. This indicates that stirring-induced dynamic SWJs account for ~11.89% absolute degradation, corresponding to ~21% of the total degradation observed under illumination. Considering that the power consumptions of stirring and illumination are 15 and 400 W, respectively, this contribution was achieved with only ~3.75% of the energy input, highlighting the favorable energy efficiency of SWJ catalysis.

Subsequently, we performed dynamic-SWJ-stimulated CEC in high-ion-concentration solutions. Figure 3D shows that FEP-based CEC can hardly degrade MO dissolved in 1 M NaCl aqueous solution, likely due to the reduced contact-electro activity under high ion concentrations, which weakens interfacial charge transfer [32]. In contrast, p-Si can still completely degrade MO. The detailed results are elaborated in Fig. S17. These findings suggest that SWJ-stimulated CEC in semiconductors can better tolerate ionic screening effects, making it suitable for high-salinity environments. This result highlights the superior salt tolerance catalysis induced by dynamic SWJs, which is critical for practical applications such as seawater treatment and high-salinity wastewater remediation. To further contextualize performance, Table S1 compares degradation efficiency, turnover number, and energy consumption across representative photocatalytic, Fenton, and piezo-phototronic systems, highlighting the advantages of the dynamic SWJ approach. In addition, the degradation of high-concentration (10, 20, and 50 ppm) MO aqueous solutions was examined. FEP exhibits a substantial decrease in degradation efficiency at higher concentrations (Fig. 3E). However, the impact on the degradation efficiency of p-Si is much smaller.

### Catalytic hydrogen evolution via dynamic SWJs

**Fig. 4.**
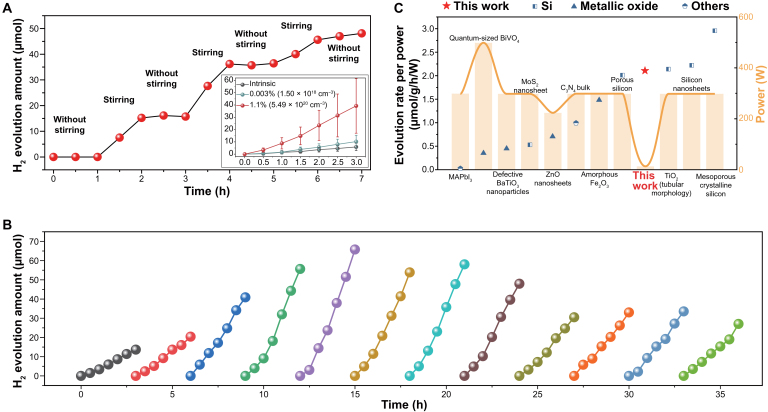
Hydrogen production via dynamic SWJs. (A) Hydrogen evolution during alternate stirring and static conditions. The inset shows the hydrogen evolution test of silicon powder with different doping concentrations. Error bars represent the standard deviation based on 3 replicate measurements. (B) Cyclic performance test of hydrogen evolution. (C) Chart comparing the hydrogen evolution under unit catalyst weight and energy consumption via dynamic-SWJ-stimulated contact-electro-catalysis (CEC) and photocatalysis.

To demonstrate the versatility of dynamic-SWJ-stimulated CEC, we conducted hydrogen evolution experiments using p-Si powder and 20% methanol solution under vacuum and dark conditions. Methanol and water are found to have differing effects on the band bending of SWJs. Specifically, methanol, as a known hole-trapping agent [33], can induce a larger band bending effect, while pure water causes less bending (Fig. S18). Therefore, the hydrogen generation process is described as follows: when the mixture of methanol and water contacts p-Si, holes transfer from p-Si to methanol, and thus, electrons transfer to p-Si; if mechanical perturbations are turned on, the proportions of methanol and water change, and when more water accumulates, the band bends upward, and electrons in p-Si will flow back to the water side, making hydrogen ions capture electrons and turn into hydrogen; and when more methanol accumulates, the band bends downward, holes will again transfer to the methanol, with electrons transferring to p-Si, forming a working period. As a demonstration, we performed the experiment for 7 h, with the stirring condition switching on and off alternatively. It was observed that hydrogen evolved only when stirring was on, as shown in Fig. 4A. As shown in the inset of Fig. 4A, the doping concentration of p-Si influences the hydrogen evolution rate, indicating that electronic property optimization could further enhance performance. The stability of hydrogen evolution in dynamic-SWJ-stimulated CEC was also tested using p-Si with a doping concentration of 1.1%. It is found that even after 12 cycles (3 h for each cycle), hydrogen evolution is still observed (Fig. 4B). The fluctuation of hydrogen evolution rate could be ascribed to the instability of the mechanical forces. Figure 4C compares the hydrogen evolution rate per catalyst gram and per energy unit for dynamic-SWJ-stimulated CEC and photocatalysis. It reveals that SWJ-stimulated CEC has a significantly low energy consumption compared to photocatalysis. Detailed results can be found in Table S2. This low energy demand makes the SWJ-based hydrogen evolution strategy highly attractive for decentralized hydrogen production, especially in off-grid or resource-limited settings. By harvesting ambient mechanical energy, such as vibrations or fluid motion, this platform may be further developed for distributed energy systems, contributing to carbon neutrality and the Sustainable Development Goals [34]. We expect that further investigations on material modifications (e.g., energy band engineering and doping concentration adjustment) and optimization of mechanical energy input will greatly improve the hydrogen evolution rate.

## Conclusion

This study reports a unique catalysis stimulated at a dynamic SWJ interface. Under mechanical stimulus, the energy band in the SWJ keeps bending forth and back, resulting in electrons and holes transferring between the semiconductor and water and therefore enabling catalytic process. We confirmed that CEC is the dominant mechanism, and this phenomenon is also feasible for various semiconductors. Moreover, SWJ-stimulated CEC can also be employed for hydrogen evolution, showing a low energy consumption. We expect that further studies on carrier transport, interfacial band bending, and material optimization in SWJs will significantly improve the catalytic rate. These advances may also extend SWJ-stimulated CEC to broader environmental and energy-related applications. Moreover, we envision that SWJ-stimulated CEC not only could enable an innovative catalytic mechanism for semiconductors but also provides a complementary strategy for photocatalysis and electrocatalysis, especially when the energy input is limited.

In addition, the generation of ROS through dynamic-SWJ-induced catalysis may open opportunities beyond chemical conversion. Given the well-established roles of ROS in biomedical processes such as antimicrobial defense and cancer treatment, this mechanism could potentially be adapted for flexible or wearable therapeutic platforms. By harvesting subtle mechanical motions (e.g., body movements), localized ROS production might be leveraged for targeted biomedical interventions, offering a conceptual route toward self-sustained, power-free therapeutic systems. Nevertheless, further dedicated biomedical studies will be required to validate and translate this potential into practical applications.

## Materials and Methods

### Chemical reagents and materials

The following chemicals were used in the experiments: FEP (Dupont), silicon dioxide (SiO_2_, Aladdin, 99.95%), *p*-benzoquinone (C_6_H_4_O_2_, Macklin, ≥99.5%), *tert*-butanol (C_4_H_10_O, Macklin, 98.0%), silver nitrate (AgNO_3_, Macklin, 99.8%), sodium sulfite anhydrous (Na_2_SO_3_, Macklin, 98.0%), TEMPO (C_9_H_18_NO, Macklin, 98%), TEMP (C_9_H_17_NO·HCl, Dojindo), DMPO (Dojindo, 99%), WST Assay Kit S311 (Dojindo), *p*-phthalic acid (C_8_H_6_O_4_, Macklin, 99%), sodium phosphate tribasic dodecahydrate (Na_3_PO_4_, Aladdin, 99.99%), sodium chloride(NaCl, Macklin, 99.5%), and MO (C_14_H_14_N_3_NaO_3_S, Macklin, 98%). p-type doped (boron doping) and n-type doped silicon (phosphorus doping) were custom-produced through the supplier.

### Sample preparation

Five milligrams of MO was added to 1 l of ultrapure water to prepare a 5 ppm MO aqueous solution. FEP, SiO_2_, p-Si, and n-Si were each prepared in 100 mg quantities and positioned in 10-ml glass tubes containing a 5 ppm MO solution. The glass tubes were covered with tin foil externally to shield them from ambient natural light. For magnetic stirring experiments, equivalent volumes were instead used in flat-bottomed glass bottles. The THA solution was prepared by combining 332.4 mg of *p*-phthalic acid with 760 mg of sodium phosphate tribasic dodecahydrate. The WST-1 solution was prepared by diluting 1 ml of WST-1 solution obtained from a Dojindo assay kit with 19 ml of buffer solution and adding 30 ml of ultrapure water. The solutions, TEMP, TEMPO, and DMPO, utilized for ERP testing were prepared as 100 mM solutions and placed in glass test tubes. Subsequently, 100 mg of p-Si was introduced into each solution. The testing procedure involved subjecting the mixtures to a 30-min evaluation using a vibrational motor, with samples being extracted during the experiment phase.

### Sample characterization

The UV–Vis absorbance of the MO solution was measured using a Shimadzu UV-3600 UV–Vis spectrometer. During Mott–Schottky testing, a dual-electrode system was employed using an electrochemical workstation (CHI660e). The working electrode featured an Ag/AgCl electrode to mitigate interface capacitance. The backside of the silicon wafer employed a gallium–indium eutectic liquid metal connection to establish ohmic contact conductors. Photodegradation was carried out using a solar simulator (model 94023A, Newport) with an AM 1.5G spectrum distribution calibrated against an NREL reference cell to accurately simulate the full solar intensity (100 mW·cm^−2^). XPS data were acquired by a Kratos AXIS Ultra DLD XPS instrument, using an Al Kα source (*hν* = 1,486.68 eV) for illumination. XPS binding energy was calibrated using adventitious alkyl carbon signals, shifting the C 1s peak to 284.8 eV. Scanning electron microscopy images of the samples were obtained using FEI Nova 450. Energy-dispersive x-ray analysis was conducted on FEI Nova 450 equipped with an AMETEK Octane Super appendix. LC–MS analysis was conducted using a Thermo Scientific Q Exactive Orbitrap Quadrupole-Electrostatic Field Orbitrap High-Resolution Tandem Mass Spectrometer. EPR was conducted with Bruker EMX plus-9.5/12/ P/L. The measurements were conducted in the X-band (9.830243 GHz), with an amplitude modulation of 1 G, a microwave power of 2 mW, an amplitude modulation frequency of 100 kHz, a conversion time of 60.00 ms, and a time constant of 40.96 ms. The emission spectra of THA–OH were measured on FLS 980 (Edinburgh Instruments), using *λ*_excitation_ = 225.0 nm. The current was collected using Stanford Research Systems model SR570 and converted to a computer-readable input/output signal using NI-USB-6259. The voltage was supplied externally by a dc power supply. The hydrogen evolution experiments were conducted using the Labsolar 6A system, and the generated hydrogen was analyzed by gas chromatography.

## Data Availability

The data supporting the findings of this study are reported in the main text or the Supplementary Materials. Raw data can be obtained from the corresponding authors upon reasonable request.

## Supplementary Materials

Supplementary 1 Figs. S1 to S23 Supplementary Notes S1 and S2 Tables S1 and S2 Movies S1 to S3 References [39–60]
