# Supplementary material for: Contact-electro-catalysis at Dynamic Semiconductor–Water Junctions
Source: Research (Wash D C). 2025 Oct 7;8:0940. doi: 10.34133/research.0940 (PMC12501637; doi:10.34133/research.0940)
Supplement: Supplementary 1 — Figs. S1 to S23 Supplementary Notes S1 and S2 Tables S1 and S2 Movies S1 to S3 References [39–60] [file research.0940.f1.zip › 5 Supplementary Materials-RESEARCH 0819.docx]

Supplementary Materials for

**Contact-electro-catalysis at Dynamic Semiconductor-Water Junction**

**Authors**

Zhanqi Liu^1,2,^ ^†^, Ziming Wang^2,3,^ ^†^, Kaiyang Shi^2,3,^ ^†^, Huifan Li^2,3^, Yusen Su^2,3^, Weihua Han^1,*^, Zhong Lin Wang^2,3,*^, Wei Tang^2,3,*^

**Affiliations**

1 School of Physical Science and Technology, Lanzhou University, Lanzhou 730000, China.

2 Center for High-Entropy Energy and Systems, Beijing Institute of Nanoenergy and Nanosystems, Chinese Academy of Sciences, Beijing 101400, China.

3 School of Nanoscience and Engineering, University of Chinese Academy of Sciences; Beijing, 100049, China.

^*^Address correspondence to: Weihua Han; [hanwh@lzu.edu.cn](https://d.docs.live.net/faf3526eb5bd260d/桌面/投稿/Research/hanwh@lzu.edu.cn), Zhong Lin Wang; [zhong.wang@mse.gatech.edu](https://d.docs.live.net/faf3526eb5bd260d/桌面/投稿/Research/zhong.wang@mse.gatech.edu), and Wei Tang; [tangwei@binn.cas.cn](mailto:tangwei@binn.cas.cn)

†These authors contributed equally to this work


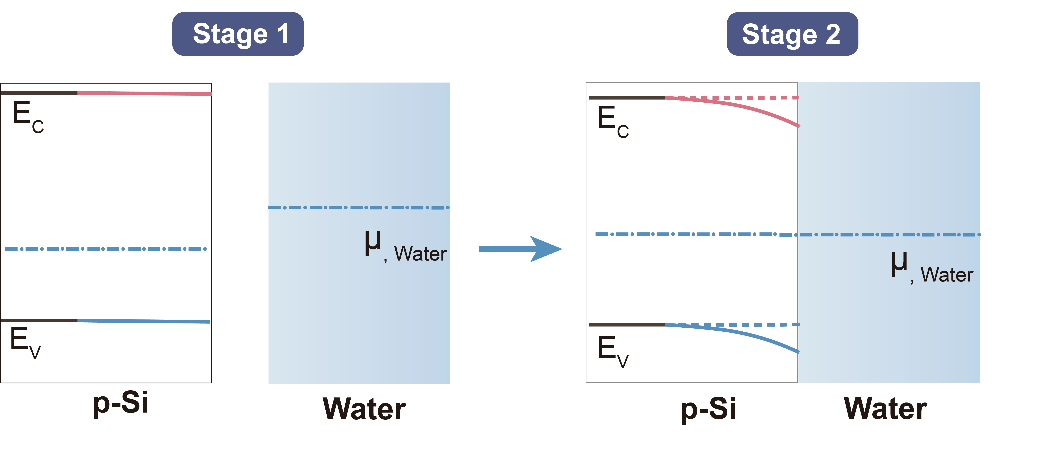


**Fig. S1.** Diagram of the band bending from the initial contact.

**Fig. S2.** **The EPR of TEMP and DMPO at different time intervals.** (A) TEMP; (B) DMPO.

**Fig. S3. Fluorescence and absorbance spectroscopy.** (A) Fluorescence spectra of THA and (B) absorption spectra of WST-1 from the sampled reaction. (C) Evaluation of hydroxyl and superoxide radicals.

**Fig. S4.** Comparison of MO degradation with and without vibration, the inset shows photographs of MO aqueous solution samples taken from 0 to 120 minutes.

**Fig. S5.** The comparative analysis of MO degradation efficiency in various sizes of p-Si.

**Fig. S6.** Further analysis of (A) the UV-Vis spectrum and (B) the LC-MS spectrum.

**Fig. S7. X-Ray Photoelectron Spectroscopy Analysis.** (A) Full Spectrum, (B) C 1s, (C) O 1s, and (D) Si 2p High-Resolution Spectrum of p-Si before and after reaction.

**Fig. S8.** Morphological characterization and Energy Dispersive X-ray (EDX) analysis of the p-Si powders before and after the reaction.


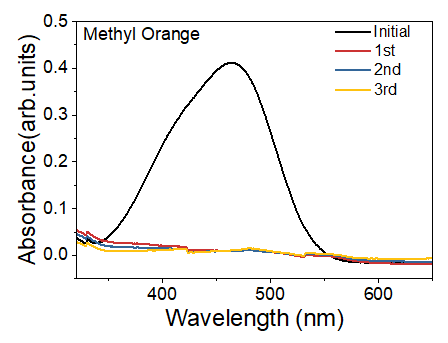


**Fig. S9.** UV–Vis spectra of 10 mL aqueous MO solution (5 ppm) before reaction and after three consecutive 120-minute degradation cycles using 100 mg p-Si powder under vibrational conditions.

**Fig. S10.** UV–Vis absorption spectra of Rhodamine B (A), methylene blue (B), and malachite green (C) aqueous solutions (5 ppm) before (0 min) and after (120 min) degradation by p-Si powder under vibrational conditions. The insets show corresponding photographs of the dye solutions before and after treatment.

**Fig. S11. Mott-Schottky plot of n-Si wafers in contact with deionized water.** (A) High doping concentration (Resistivity < 0.02 Ω·cm); (B) Medium doping concentration (Resistivity: 1~20 Ω·cm); (C) Low doping concentration (Resistivity > 1000 Ω·cm).

**Fig. S12.** Evolution of n-Si structures with water over time obtained from AIMD simulations. The snapshots show the system configurations at specific time points, illustrating the dynamic changes in the hydrogen bond network and the interaction of water molecules with the n-Si. The configurations are taken at 0, 1, 3, 6, 10 ps. The blue, green, red, and pink spheres represent silicon, boron, oxygen, and hydrogen atoms, respectively. The hydrogen atom marked in sky blue represents the active protonic hydrogen.

**Fig. S13.** Detailed UV-Vis spectra result of MO degradation by various semiconductor: (A) n-Si, (B) GaN, (C) Fe_2_O_3_, (D) InSb, (E) Ag_2_O, (F) WO_3_, (G) AgS, (H) CuO.

**Fig. S14.** **The detailed degradation process of MO concentration over time under different conditions.** Comparison of MO degradation among FEP, P-Si, and SiO_2_ under (A) ultrasonic, (B) vibration, and (C) stirring conditions. (D) Comparison of MO degradation under ultrasonic conditions between p-Si and FEP in the presence of 1M NaCl. The influence of elevating MO concentrations on the degradation of MO by (E) p-Si and (F) FEP under ultrasonic conditions.

.

**Fig. S15. Comparative analysis of degradation efficiency at different stirring speeds and LC-MS testing.** (A) Degradation of MO under magnetic stirring at different rotation speeds. (B) Mass spectrometric analysis of MO under magnetic stirring conditions at 1500 rpm, following separation by liquid chromatography at different reaction times.

**Fig. S16.** Comparison of MO degradation among n-Si, p-Si, FEP and SiO_2_ under simultaneous stirring and lighting conditions.

**Fig. S17.** The detailed results of MO degradation in the presence of 1M NaCl. (a) p-Si, (b) FEP.


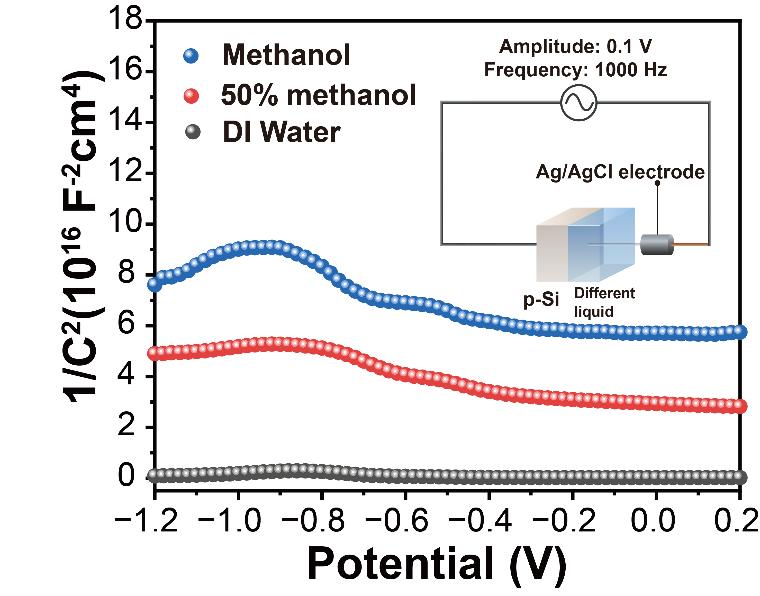


**Fig. S18.** Mott-Schottky test at different methanol concentrations.

**Fig. S19. Real-time monitoring of surface current during dynamic water contacts with p-type silicon.** (A) Schematic diagram of the experimental procedure. (B) Current-voltage testing and (C) Mott-Schottky plot of the semiconductor surface. (D) Current-time testing under a 1.5 V DC bias applied to the electrodes and an injection rate of deionized water at 0.05 mL/s (The blue shaded area represents the start of droplet dripping, the yellow shaded area represents the stop of droplet dripping, and the inset shows the conduction mode in the steady state). (E) Enlarged view of the start of droplet dripping. (F) Enlarged view of a single cycle of droplet dripping. The inset illustrates the current conduction mechanism during hole transfer and space charge layer formation. (G) Enlarged view of the stop of droplet dripping (The yellow shaded area represents air injection; the inset shows the current decrease mechanism during air injection).

**Fig. S20. Real-time monitoring of surface current during dynamic water contacts with p-type silicon under different conditions**: (A) V_bias_=1.5 V, IR=0.01 mL/s. (B) V_bias_=1.5 V, IR=0.05 mL/s. (C) V_bias_=1.5 V, IR=0.1 mL/s. (D) V_bias_=0.5 V, IR=0.05 mL/s. (E) V_bias_=1 V, IR=0.05 mL/s. (F) V_bias_=2 V, IR=0.05 mL/s. The insets in each image are enlarged views under their respective stable conditions.

**Fig. S21.** Evolution of p-Si structures with water over time obtained from AIMD simulations. The snapshots show the system configurations at specific time points, illustrating the dynamic changes in the hydrogen bond network and the interaction of water molecules with the p-Si. The configurations are taken at 0, 1, 3, 5.5, 6.5, 12 ps. The blue, green, red, and pink spheres represent silicon, boron, oxygen, and hydrogen atoms, respectively. The hydrogen atom marked in sky blue represents the active protonic hydrogen.

**Fig. S22.** The evolution of the p-Si/water system over time after an O_2_ molecule was randomly introduced obtained from AIMD simulations. The snapshots show the system configurations at specific time points, illustrating the dynamic changes in the hydrogen bond network and the interaction of water molecules with the p-Si. The configurations are taken at 0, 0.7, 1.4, 2.5, 3, 3.5,3.8, 4.5, 5 ps. The blue, green, red, and pink spheres represent silicon, boron, oxygen, and hydrogen atoms, respectively. The hydrogen atom marked in sky blue represents the active protonic hydrogen. The introduced oxygen atoms are marked in orange to distinguish them from the oxygen atoms in water.


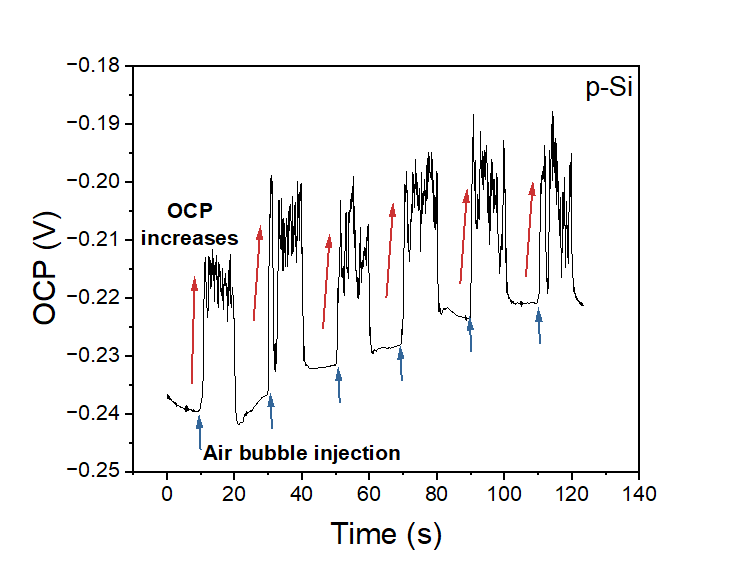
**Fig. S23.** Time-resolved open-circuit potential (OCP) response of the p-Si-water junction under bubble perturbation.

**Note S1:**

In the main text, we propose the dynamic semiconductor-water (SW) junction induced catalysis mechanism upon the contact of p-Si powder with water. We also utilized electron spin resonance spectroscopy to observe the formation of radicals in water. To address the unclear aspects regarding carrier transfer mechanisms in the dynamic SW junction mechanism of p-Si, we devised a method for real-time observation of surface currents to validate.

**Section S1. Mechanisms of Charge Transfer in p-Type Silicon During Water Droplet Contact**

On the surface of silicon wafer, gallium indium eutectic was connected, and electrodes were drawn out. The silicon wafer is heavily p-type doped along the <100> crystal orientation, exhibiting a resistivity within the range of 0.001 to 0.005 Ω·m. A direct current voltage was applied between the electrodes, while deionized water droplets were dispensed at a specific injection rate (IR) between them. The schematic diagram of the testing method is depicted in Fig. S19A. We conducted current-voltage and Mott-Schottky testing of the semiconductor surface, as depicted in Fig. S19B and 17C. In the lower voltage range, liquid metals can also readily form Schottky junctions with silicon wafers, so it is advisable to conduct testing at higher voltages, such as 1.5 V, marked with thick black dots. Under a bias voltage (V_bias_) of 1.5 V and an injection rate of 0.05 mL/s, real-time monitoring of current was conducted, as illustrated in Fig. S19D. During the initial dripping stage (blue shaded area), we observed a stepwise increase in current, with the enlarged image depicted in Fig. S19E. The current exhibits this stepwise pattern with a period of 0.7 s. Upon detailed observation of a single cycle of current, it is evident that the current initially rises and then declines, as depicted in Fig. S19F. First, we can ascertain that the rising current results from the instantaneous contact between the water droplet and the silicon wafer. The transient rise in current, which is not sustained, suggests the presence of non-equilibrium charge carriers induced by the water droplet. These carriers are swept towards the electrodes by the lateral voltage, leading to the observed increase in current. When the charge carriers are depleted, a space charge layer forms, leading to an increase in surface resistance and consequently a reduction in current. This process should also be transient. However, during prolonged current testing, the current initially shows a gradual increase, followed by relative stability, rather than remaining consistently stable throughout (Fig. S19D). The gradual decrease in resistance and its eventual stabilization arises from the formation of an electrical double layer at the semiconductor-solid/liquid interface, facilitating the adsorption of ions. The gradual decrease in resistance and its eventual stabilization results from the formation of an electrical double layer at the semiconductor-solid/liquid interface, which is caused by the adsorption of ions (The inset in Fig. S19D). Even in the state of ion adsorption, the transient increase in current upon instantaneous droplet contact can still occur. This can also explain why p-Si powder is almost unaffected by contact electrification, maintaining a high catalytic efficiency. By distinguishing between these transient and steady-state phenomena, we can elucidate the mechanism of charge carrier transfer when a semiconductor contacts water and forms a space charge layer.

**Section S2.** **Analysis of stepwise current under different conditions**

We compared real-time monitoring of surface current under different conditions, as shown in Fig. S20. When the IR is changed, the period of the stepwise current varies (Fig. S20A, B, and C). We selected individual stepwise currents at the ion adsorption equilibrium and found that the periods were 4.47 s (IR=0.01 mL/s), 0.66s (IR=0.05 mL/s), and 0.4 s (IR=0.1 mL/s), respectively (periods denoted as T in the inset; Fig. S20B is selected from Fig. S19D). The rise in current ΔI caused by non-equilibrium hole transfer is 0.96 μA, 0.24 μA, and 0.12 μA, respectively, and it gradually decreases as IR increases. This indicates that a slower injection speed provides more time to neutralize the adsorbed ions and restore the space charge layer, leading to the generation of more non-equilibrium holes when the next droplet arrives. Under the same IR condition (IR = 0.05), ΔI increases with increasing voltage (Fig. S20D, E, B, and F). Under the same IR condition (IR = 0.05 mL/s), ΔI increases with increasing voltage (Fig. S20D, E, B, and F, with biases of 0.5 V, 1 V, 1.5 V, and 2 V), resulting in ΔI values of 0.06 μA, 0.18 μA, 0.24 μA, and 0.63 μA, respectively. Considering relative equilibrium in ion adsorption, as the voltage increases, the current demonstrates a notable increase, further supporting the existence of non-equilibrium hole transfer mechanisms. In addition, when investigating the rise time (T_rise_) of the stepwise current under these conditions, we found it to be approximately 0.2 s (0.19 s in Fig. S20 A, 0.18 s in 20B, 0.16 s in 20C, 0.23 s in 20D, 0.21 s in 20E, 0.22 s in 20F). The T_rise_ remains relatively consistent, showing minimal sensitivity to V_bias_ and IR. This indicates that the process of non-equilibrium hole transfer is instantaneous and less influenced by external conditions.

**Section S3.** **Time-resolved open-circuit-potential verification of bubble-induced band-flattening**

To provide an independent probe, we performed a time-resolved open-circuit-potential (OCP) experiment on the same p-Si–water system described in **Fig. S23**. P-type silicon wafer with a backside ohmic contact served as the working electrode, immersed in DI water and measured against an Ag/AgCl reference electrode, while mechanical perturbation was introduced by injecting air bubbles just above the silicon surface.

As illustrated in Fig. S23, arrival of the bubbles at the Si-water interface caused the OCP to move anodically from -0.240 V to -0.215 V; the +25 mV excursion vanished as soon as the bubbles burst and the surface became fully wetted again. Because an anodic shift of E_OCP_ corresponds to an equal but opposite change in the space charge potential (ΔE_OCP_ ≈ -Δϕ_b_), the measurement reveals that the downward band bending of p-Si is momentarily reduced by ≈ 25 mV, that is, the bands are transiently flattened. We attribute the flattening to electron transfer from surface states into dissolved O₂ at the bubble boundary, in combination with the temporary decrease of Helmholtz capacitance caused by the liquid-to-gas replacement. The amplitude of the OCP shift closely matches the 30 mV flat-band displacement obtained from the Mott–Schottky analysis in Fig. 1B, providing an independent, time-resolved verification of the dynamic semiconductor-water-junction mechanism proposed in the main text.

**Note S2:**

Ab initio molecular dynamics (AIMD) simulations were conducted using the Vienna Ab initio Simulation Package (VASP).[39,40] Electron-ion interactions were modeled with projector-augmented wave (PAW) pseudopotentials,[41] employing a cutoff energy of 400 eV. The Perdew−Burke−Ernzerhof generalized gradient approximation (GGA-PBE)[42] was employed for the exchange-correlation functional, with a Gaussian smearing width of 0.05 eV. Convergence criteria for electronic self-consistent iteration were set to 1×10^−5^ eV. Van der Waals (vdW) interactions were included using the DFT-D3 method.[43]

To model the p-Si and n-Si slabs, boron and phosphorus atoms replaced two silicon atoms on an eight-layer 2 × 2 Si (100) surface. Interfaces of P-Si/water and N-Si/water were simulated by introducing 20 water molecules above each respective slab. The water film and vacuum layer had approximate thicknesses of 1 nm and 2.5 nm, respectively. The simulation box dimensions along the z-axis were approximately 4.4 nm.

To expedite AIMD simulations, classical molecular dynamics was employed initially to equilibrate hydrogen bond networks within the water layer. The p-Si/water and n-Si/water configurations were equilibrated at 298 K under the NVT (canonical) ensemble for 12 ps. Subsequently, an O_2_ molecule was randomly introduced to the water/vacuum interface of the equilibrated p-Si/water system. The system was further equilibrated at 298 K under the NVT ensemble until H_2_O_2_ production was observed.

**Section S1. Evolution of p-Si contact with water.**

Based on the simulation results, it is observed that upon contact between water and the p-Si system, adsorption occurs initially even in the absence of external influences (3 ps), as shown in Fig. S21. Subsequently, the hydrogen protons from the adsorbed water molecules transfer to other water molecules (5.5 ps). This leads to the formation of hydroxyl radicals on the p-Si surface, consistent with stage 1 described in Figure. 1A of the main text. The results at 6.5 ps and 12 ps indicate that hydrogen protons remain present in the water. This simulation demonstrates that when p-Si contacts water, electron exchange occurs, leading to the formation of hydroxyl radicals. The entire process can be seen in Movie S1. positively charged hydrogen protons, present as hydrated hydrogen ions in water, reach equilibrium with the electron-rich surface of p-Si.

**Section S2.** **Evolution of p-Si/water systems after reaching equilibrium and O_2_ introduction.**

Based on the equilibrium reached in the p-Si/water system shown in Fig. S21, oxygen molecules were introduced (Fig. S22). Within the 0-3 ps time range, oxygen molecules gradually enter the water. At 3.5 ps, an oxygen molecule combines with a hydrogen proton, and an additional water molecule is adsorbed onto the p-Si surface. Subsequently, the hydrogen atom of the adsorbed water molecule enters the water, forming a new hydrated hydrogen ion (3.8 ps). At this point, the OOH group forms, accompanied by electron transfer, and the Si surface reaches equilibrium. Subsequently, new water molecules can continue to adsorb onto the Si surface, proceeding with the subsequent steps. The hydrated hydrogen ion continues to transfer a proton to the oxygen molecule, which combines with a hydrogen proton (4.5 ps), eventually forming a hydrogen peroxide molecule at 5 ps. The entire process can be seen in Movie S2. The oxygen molecule forms neutral hydrogen peroxide, and the hydroxyl radicals adsorbed on the p-Si surface are also neutral. Since the entire system is charge balanced, the electron-rich p-Si surface is neutral as well. Thus, the addition of oxygen balances the electrons on the p-Si surface and further leads to the formation of hydroxyl radicals, which is consistent with stage 2 in Figure. 1A of the main text.

**Section S3. Evolution of n-Si contact with water**

When water contacts n-Si, water molecules gradually adsorb onto the n-Si surface within the 0-3 ps time range, as shown in Fig. S12**.** However, unlike with p-Si, the hydrogen atom of the adsorbed water molecules does not immediately enter the water. The hydrogen atoms oscillate back and forth between the adsorbed water and other water molecules (6 ps and 10 ps). This is due to the properties of n-Si. Unlike p-Si, the different doping type results in fewer hydroxyl radicals forming when n-Si contacts water. The entire process can be seen in Movie S3.

**Table S1.** Performance comparison of mainstream catalytic systems for pollutant degradation.

| Catalysts | Features | Condition | Degradation Efficiency (%) | TON  (μmol/mol) | Energy Consumption (kWh/m³) | Energy per mole (kWh·mol⁻¹) | Ref. |
| --- | --- | --- | --- | --- | --- | --- | --- |
| TiO_2_ | photocatalysis | 250 W Xe lamp, pH = 3 | 100% in 300 min | 719 | 1500.0 | 3.75×10^4^ | 44 |
| BiVO₄ microtubes | photocatalysis | 250 W lamp + 10 mM H₂O₂ | 95% in 180 min | 140 | 75000 | 1.23×10⁶ | 45 |
| ZnO:Eu | photocatalysis | 300 W lamp | 100% in 60 min | 2490 | 30000 | 1.96×10⁵ | 46 |
| Fe⁰/H₂O₂ | Advanced Fenton | 125 W UV | 100% in 15 min | 34200 | 156.25 | 5.12×10³ | 47 |
| Fe₂O₃–SiO₂/ H₂O₂ | photo-Fenton | 1000 W halogen lamp | 97% in 30 min | 24800 | 5000 | 1.64×10⁵ | 48 |
| CdS-Au (4%) | Piezo-phototronic | 300 W Xe lamp + ultrasound (45 kHz) | 100% in 15 min | 3530 | 750 | 6.14×10⁴ | 49 |
| p-Si | Contact-electro-catalysis | 0.21 W mechanical vibrations | 100% in 120 min | 43 | **42** | ≈2.7×10³ | This work |

**Table S2.** Hydrogen evolution rates and energy source of commonly used photocatalytic materials and their comparisons with this study.

| **Type** | **Catalyst** | **Energy source** | **Evolution rate (μmol/g/h)** | **Evolution rate per power**  **(μmol/g/h/w)** | **Ref** |
| --- | --- | --- | --- | --- | --- |
| Si | Porous silicon | 300 W, Xe lamp | 604.7 | 2.02 | 50 |
|  | Silicon nanosheets | 300 W, Xe lamp | 723 | 2.41 | 51 |
|  | Mesoporous crystalline silicon | 300 W, Xe lamp | 882 | 2.94 | 52 |
| Metallic oxide | Quantum Sized BiVO_4_ | 500 W, Xe lamp | 186 | 0.372 | 53 |
|  | Defective BaTiO_3_ Nanoparticles | 300 W, Xe lamp | 132.4 | 0.44 | 54 |
|  | ZnO Nanosheets | 225 W, Xe lamp | 160 | 0.71 | 55 |
|  | Amorphous Fe_2_O_3_ | 300 W, Xe lamp | 449.5 | 1.498 | 56 |
|  | TiO_2_ (multilayered tubular morphology ) | 300 W, Xe lamp | 672 | 2.24 | 57 |
| Others | MAPbI_3_ particles | 300 W, Xe lamp | 14 | 0.05 | 58 |
|  | MoS_2_ nanosheet | 300 W, Xe lamp | 156 | 0.52 | 59 |
|  | C_3_N_4_ bulk | 300 W, Xe lamp | 300 | 1.00 | 60 |
| This work | p-Si | 12W, magnetic stirrer | 26.7 | 2.22 | This work |
